# Epithelial vertex models with active biochemical regulation of contractility can explain organized collective cell motility

**DOI:** 10.1063/1.5023410

**Published:** 2018-07-23

**Authors:** Sarita Koride, Andrew J. Loza, Sean X. Sun

**Affiliations:** 1 Chemical and Biomolecular Engineering, Johns Hopkins University, Baltimore, Maryland 21218, USA; 2 Department of Cell Biology, Washington University School of Medicine, St. Louis, Missouri 63110, USA; 3 Mechanical Engineering, Johns Hopkins University, Baltimore, Maryland 21218, USA; 4 Institute of NanoBioTechnology, Johns Hopkins University, Baltimore, Maryland 21218, USA

## Abstract

Collective motions of groups of cells are observed in many biological settings such as embryo development, tissue formation, and cancer metastasis. To effectively model collective cell movement, it is important to incorporate cell specific features such as cell size, cell shape, and cell mechanics, as well as active behavior of cells such as protrusion and force generation, contractile forces, and active biochemical signaling mechanisms that regulate cell behavior. In this paper, we develop a comprehensive model of collective cell migration in confluent epithelia based on the vertex modeling approach. We develop a method to compute cell-cell viscous friction based on the vertex model and incorporate RhoGTPase regulation of cortical myosin contraction. Global features of collective cell migration are examined by computing the spatial velocity correlation function. As active cell force parameters are varied, we found rich dynamical behavior. Furthermore, we find that cells exhibit nonlinear phenomena such as contractile waves and vortex formation. Together our work highlights the importance of active behavior of cells in generating collective cell movement. The vertex modeling approach is an efficient and versatile approach to rigorously examine cell motion in the epithelium.

## I. INTRODUCTION

Organized motion of epithelial cells as a group is crucial to developmental processes such as embryo patterning and organ formation.^1–3^ Epithelia are tissues that form the surface for most organs in the body. They are broadly classified into simple monolayered and stratified (multi-layered) tissues. Depending on the shape of cells in the tissue, the simple monolayer epithelium can be further classified as squamous (flat cells), cuboidal (can take on any shape), or columnar (long, column like). Irrespective of the type of epithelium, cells are connected to each other through three kinds of junctions: tight junctions,^4^ adherens junctions,^5^ and desmosomes.^6^ Adherens/tight junctions are the primary force transducers between cells while the desmosomes act as barriers to flow of water and proteins between cells. Coordinated motion of cells in an epithelial sheet is facilitated by forces (coordinated or random) generated by cells, as well as cell-cell mechanical interactions.

During organ formation or embryogenesis, coordinated cell movements and rearrangements can generate complex organ shapes. Examples include tissue folding and bending during gastrulation,^7,8^ convergent extension^9^ during tissue elongation, and neural tube formation.^10^ Forces acting on cells play a key role in shaping a tissue.^11,12^ These forces could be a result of intrinsic elasticity of a cell; from its tendency to resist stretch, or from cells exerting forces on each other through adherens junctions.^13^ These forces could also result from contractile forces coming from molecular motors.^14^ Based on these forces and measurements of cell movement, we can model the kinematics and dynamics of the epithelial sheet. Force landscape underlying a migrating monolayer has been mapped out and is shown to be rugged and heterogeneous. Plithotaxis, defined as the tendency for each individual cell within the monolayer to migrate along the local orientation of the maximal normal stress or minimal shear stress, is a newly discovered mode of cell guidance which requires force transmission across cell-cell junctions.^15^ In wound healing or tumor invasion, where cells move to cover unfilled gaps, Kim *et al.* showed another model of cell guidance where the cells at the edge exert tractions that pull systematically towards the gap using monolayer stress microscopy.^16^ In another similar biological context of filling gaps, Rodríguez-Franco *et al.*^17^ discovered propagation of deformation waves across the monolayer during boundary formation between two cell sheets. They also showed that contractility plays a role in wave generation by inhibiting tension in the monolayer.

Modeling epithelial cell dynamics can help us validate competing hypotheses and design further experiments to gain a better understanding of collective cell motility and organ formation. There are several models in place to understand sheet dynamics. These could be broadly classified into continuum and discrete models. Continuum models consider the whole cell sheet to be a two dimensional compressible fluid.^18^ Discrete models consider cells as particles^19^ and model their behavior based on forces acting on them. These discrete models can further be classified into lattice models,^20–22^ cell-centered models,^23–26^ and vertex models.^27–29^ Lattice based models have been widely used to understand cell migration on patterns.^31^ Cell shape as a function of RhoGTPase distribution has also been modeled in single cells using lattice based models.^32^ Cell centered models focus on establishing forces between cell-centers, whereas, in vertex models, each cell is modeled as a polygon representing the cell membrane. Each cell vertex has an equation of motion which depends on its connections to other vertices and the properties of its neighbors. Vertex models, in comparison to cell centered models, can more easily describe nonconvex shapes and cell neighbor rearrangements. However, these models can be computationally more expensive, especially when representing cells more accurately in 3D. Certain topological changes like cell-cell fusion, cell extrusion can be challenging to represent. In these models, it is assumed that three cells share a vertex which is not always true. Also, the cell-cell junction is assumed to be linear and unique. In reality, these are two different cell membranes that can be regulated independently.

Vertex models were first used to understand dynamics of soap bubbles and foams.^30,33,34^ These were later used to study epithelial dynamics, first by Honda and Eguchi.^35–37^ Since then, many studies used similar models^38,39^ for their ease of analysis to study cell packing and motion during morphogenetic events. Despite the prevalence of these models in studying epithelial dynamics, very few studies incorporate the role of cell signaling into these primarily mechanical models.^40–43^ Many experimental studies have shown that cells actively adjust their forces in response to externally applied forces. These forces are modulated by biochemical signaling mechanisms that receive their signals from the cell membrane.^44^ Protrusion at the cell front and contraction at the rear are active processes which help the cell move and are modulated by signaling mechanisms. Protrusion involves actin filaments pushing against the cell membrane generating forces on the order of nanonewtons per micron. On a similar order, contractile forces are generated by myosin contraction in the cell cortex. In the epithelial context, cells also adjust their contractile forces, leading to phenomena such as epithelial oscillations.^45^

In this study, we combine the vertex mechanical model with active biochemical signaling control of cell forces. Studies have shown changes in activation of RhoGTPases in response to an external force.^46,47^ In addition, pattern formation of RhoGTPases has been observed during wound healing.^48,49^ RhoA is a principal mediator of cytoskeletal tension.^50^ It regulates the activity of myosin^51^ and therefore is responsible for most of the intracellular tension and forces. Active Rho propagates downstream signals by binding to Rho associated kinase, ROCK. Phosphorylation of myosin light chain by ROCK leads to contractile force generation.^52^ Manipulation of the Rho pathway in experiments has shown the importance of Rho and ROCK in regulating myosin activity and hence tension generation.^53^ In one study, inhibition of Rho kinase led to a decrease in myosin filament mass in muscle cells.^54^ In another study,^55^ using isolated reactivatable stress fibers, it was shown that Rho kinase plays a major role in maintaining sustained contraction in cells. To understand how active contractility, which is dependent on GTPase signaling, works hand in hand with cell shape changes and motion, we incorporated a Rho-Myosin signaling mechanism within our vertex model. In the Methods section, we describe the vertex model in detail. In the Results section, we describe two aspects of collective migration we studied using this model—effects of cell density and confinement. We find that cell density can modulate the level of cell contraction and influence the overall migration speed and cell-cell correlation. We also find collective emergent patterns such as contractility waves and streaming behavior, even though force generation by individual cells is not coordinated by cell-cell signaling. For confined migration, we also observe streaming behavior in the form of coherent rotations, with occasional counter rotations within the streaming group. The active contractility mechanism also generates vortical defects within the coherent streaming. These simulation results are consistent with observed collective migration phenomena observed in experiments and suggest that simple models of active cell mechanics without cell-cell communication can explain most of these phenomena.

## II. RESULTS

### A. Cell shape and speed change as a function of time in response to changes in myosin content

**FIG. 1.**
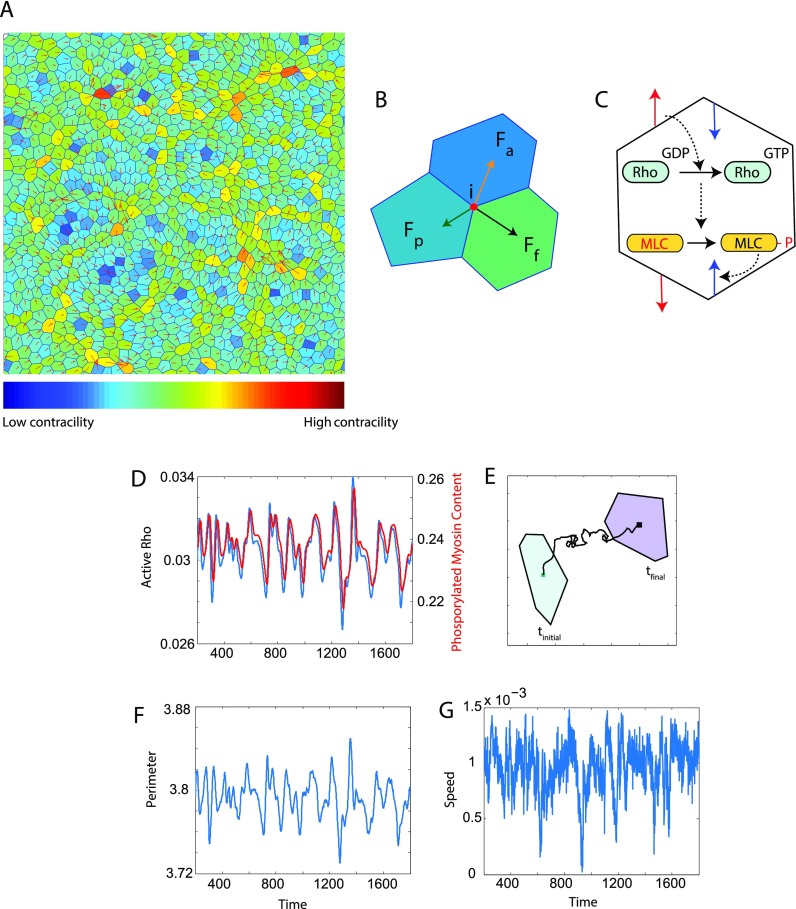
Description of the vertex model and characterization of motion of a single cell in the modeled epithelium in the presence of an actively regulated contractile force. (A) Cells in an epithelial sheet are modeled as polygons. Each polygonal vertex is shared by three cells and each edge is shared by two. The velocity of the cell is shown by the red arrows. The color of each cell represents the phosphorylated myosin content in it. Blue represents low levels of phosphorylated myosin and hence low contractility and red represents high contractility. Phosphorylated myosin content of a cell determines the magnitude of contractile force generated and hence the cell shape. (B) Motion of cells is determined by active (*F_a_*), passive (*F_p_*) and friction (*F_f_*) forces acting on all the vertices that define the cell boundary. (C) As the cell changes shape and volume, signaling via the Rho pathway alters the level of active intracellular contractility. The red arrows represent tensile forces acting on the cell to which the cell responds with contractile forces (blue arrows) via the Rho pathway. When a cell experiences a mechanical stretch due to forces from its neighbors, the Rho-Myosin pathway gets activated resulting in cell contraction. In this implementation of the vertex model, as a cell moves from one point to the other in time (here *t_initial_* to *t_final_*) as shown in (E), fluctuations in phosphorylated Rho and Myosin (D) fractions, cell perimeter (F) and speed (G) (dimensionless) are displayed.

Experiments in literature^46,47^ suggest that when a cell experiences an external stretch, it leads to activation of RhoGTPase. This activates Rho associated kinase (ROCK) which phosphorylates myosin light chain leading to cell contraction. This negative feedback loop is incorporated into our vertex model. A detailed description of our model is given in the Methods section. Briefly, each cell is modeled as a polygon with shared vertices and edges [Fig. 1(A)]. Motion of the cell arises from passive (*F_p_*, elastic and adhesive), frictional (*F_f_*), and active (*F_a_*, protrusive and contractile) forces acting on the cell vertices [Fig. 1(B)]. The contractile force is modified to incorporate dynamic signaling of Rho-ROCK-MLC activation [Fig. 1(C)] sensitive to the physical environment of each cell. As a cell moves from one point in space to another, the fraction of active Rho [Figs. 1(D) and 1(E)] and myosin fluctuates in time. The corresponding change in perimeter is shown in Fig. 1(F), and the speed of the cell is shown in Fig. 1(G). Incorporating a signaling pathway for cell contractility into the vertex model gives us a way to understand the response of the cell to different environmental constraints. We refer to this method as active contractility or contractility with signaling in the rest of the text.

### B. Effect of cell density on motility

The motion of a single cell in a confluent layer depends on the local forces acting on it. These forces depend on its connectivity to its neighbors. The average number of neighbors of a cell can be important for determining the characteristics of collective motion. In other words, cell density or the number of cells per unit area of the sheet could be an important player in determining how organized motion is at the tissue level. Several experiments have shown the importance of density on collective cell motion^62–65^ but exactly how it affects migration is not clearly understood.

To understand how cell density affects collective cell migration, we simulated cells in a square domain with periodic boundary conditions. We changed density by fixing the number of cells and varying the simulation domain. With the same number of cells, bigger box sizes simulate low density conditions and smaller box sizes represent high density conditions.

#### 1. Mean cell speed decreases with increase in density

**FIG. 2.**
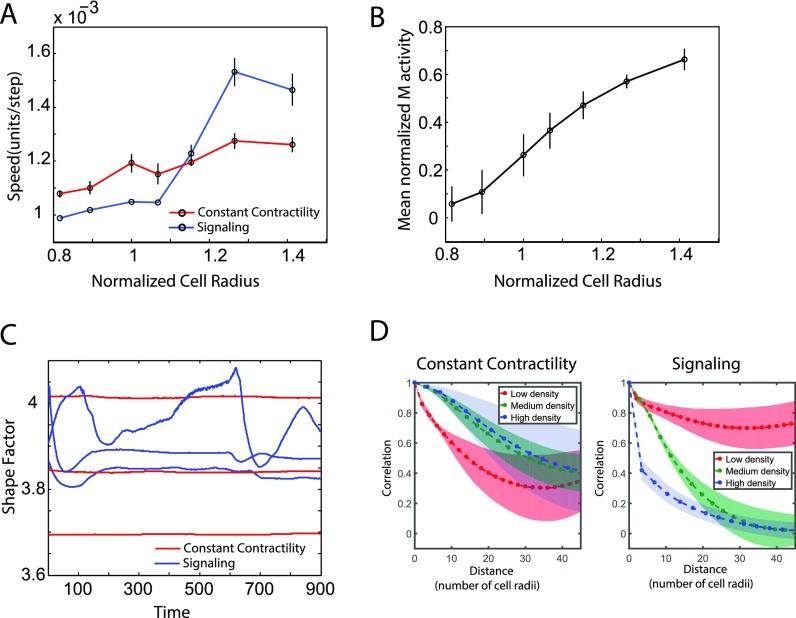
Comparison between vertex model with constant contractility and active Rho-Myosin signaling. (A) Cell speed as a function of normalized cell radius. Normalized cell radius is used as a proxy for cell density. The signaling model shows a greater change in cell speed as a function of density as compared to the constant contractility model. (B) Myosin levels in the active signaling increase as a function of cell radius. (C) Shape factor defined as a perimeter over square root of area fluctuates in time in the signaling model. (D) The radial spatial correlations of cell velocities are qualitatively different at different cell densities when a vertex model with signaling is used.

Simulations of cells at different densities were performed, and the mean cell speed was calculated. The mean cell speed is plotted as a function of normalized cell radius. Here, the cell radius is a proxy for density as it scales inversely with the cell density. The results are shown in Fig. 2(A). Here, we compared the cell speed as a function of density using the active contractility model and the model without the signaling pathway for contractility—referred to as the constant contractility model. In the constant contractility model, the contractility coefficient of a cell *J*, *T_J_* is set to a constant value (*k*) and is not proportional to the amount of phosphorylated myosin. In both cases, there is a decrease in the cell speed as the density increases as observed in experiments. The average cell speed approximately drops by 2-fold in experiments^66^ with the increase in the density. In another paper,^67^ increasing density in the same range as considered in our paper decreases the average cell velocity by about 3-fold. In our simulations, using the active contractility model, the decrease in the speed is approximately 1.6-fold for the same range of densities. The net amount of change in the cell speed at low and high densities (between the high and low mean cell radius) and the rate at which the cell speed decreases is different in the two models.

#### 2. Mean myosin levels decrease with the increase in the density

In addition to looking at cell velocities which represent physical aspects of cell behavior at different densities, we could analyze the chemical state of the cells in terms of the mean myosin levels. We chose myosin levels as we know the intrinsic contractility of the cell depends on the amount of myosin present. The signaling component of the model gives us a way of looking at effects of density on the local myosin levels and hence the contractility of a cell. In experiments, we see that the myosin levels increase linearly with a decrease in density or an increase in the effective cell radius.^66^ Mean myosin content increased approximately 4-fold in experiments when the average cell radius increased from 8 to 15 *μ*m (approximately 1.9-fold). Our model showed an increase in normalized myosin activity of approximately 6-fold on increasing normalized cell radius. RhoA activation in the signaling component of our model depends on the change in perimeter experienced by the cell due to an external force. Since the increase in the density implies a reduction in cell area/perimeter, myosin levels decrease in simulations as seen in experiments. This is consistent with our hypothesis of stretch dependent myosin activation in a cell sheet.

Shape factor defined as cell perimeter over area varies in time in the active contractility model whereas it does not change in the constant contractility model. This result is shown in Fig. 2(C).

#### 3. Active contractility model captures cell organization of migrating collectives at different cell densities

Radial spatial autocorrelation of cell velocities (defined in Sec. IV) shows different behavior at low, medium, and high cell densities as shown in Fig. 2(D). This is correctly captured using a vertex model with contractility signaling.^66^ In this figure, low density refers to the case when the average cell area is twice the preferred area, *A*_0_, medium density is when the mean cell area is equal to the preferred cell area, and the high density case is when the mean cell area is two-third of *A*_0_. The constant contractility model does not show this variation in correlations with the density. Here, the radial distance is normalized by the mean cell radius. A value of one implies that cell motion is aligned and zero implies motion is random.

### C. Persistence and contractility determine cell organization and collective behavior at various densities

A range of cell density factors (defined as the ratio of preferred cell area to mean cell area) from 0.5 to 1.5 was explored in Sec. II B. In this section, only 0.5, 1, and 1.5 are used for low, medium, and high densities, respectively. In order to understand the effect of persistence and contractility on organizational behavior of migrating cell collectives at different densities, we considered a range of values for persistence force strength *α* and contractility constant *k* as shown in Table I. Radial spatial correlation plots at different regions in the persistence—contractility—density phase space were computed. In order to understand the cell organization, these correlation plots were fitted to an exponential function given by

$$C(\rho )=(1-C_{p})e^{-\rho /\lambda }+C_{p}.$$ (1)

Here, *C*(*ρ*) is the correlation function at a distance *ρ* and *C_p_* is the correlation plateau value at large distances. Experimentally, it was observed^66^ that the velocity correlation function goes to zero approximately around *ρ* = 34 at low cell densities. *λ* is the correlation decay length. Thus, two parameters are identified from the correlation plots—correlation length (*λ*) and correlation plateau value (*C_p_*). The correlation length is a metric identifying the radial distance at which the cell motions are strongly correlated. This is a measure of local organization. When the complete epithelium migrates as a group, the correlation function does not go to zero. The correlation plateau value is a measure of this extensive streaming behavior.

**FIG. 3.**
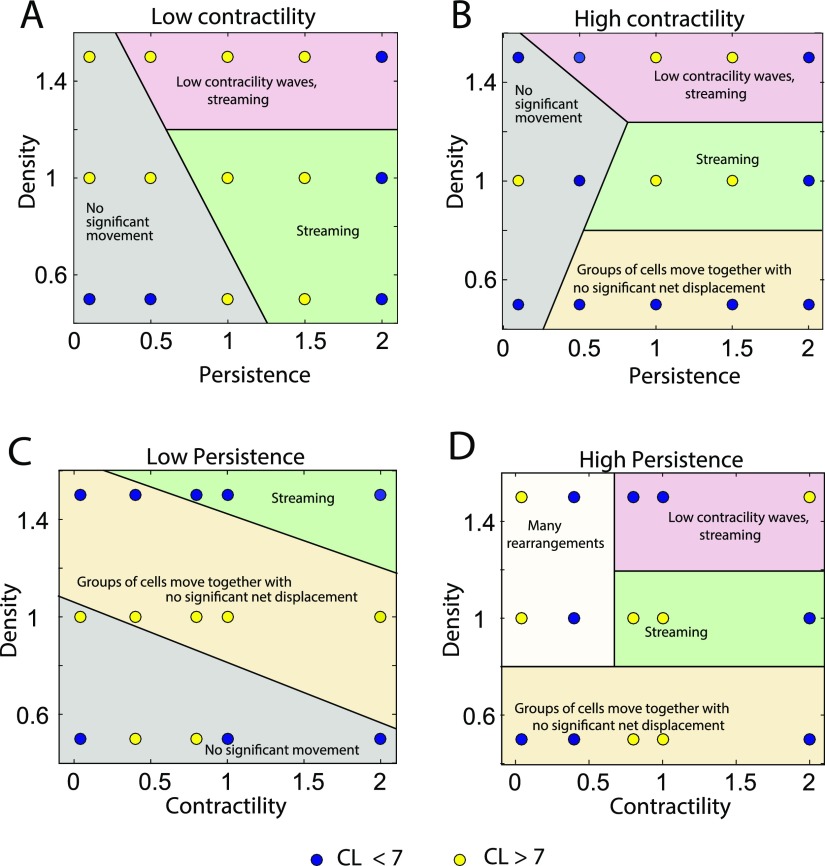
Behavior of the correlation length as a function of cell density, contractility and cell persistence force. Correlation length—low (blue), high (yellow)—at low, medium, and high densities shown at different persistence and contractility parameter values. Various cell behaviors like streaming and jammed in place are labeled in the phase plots.

**FIG. 4.**
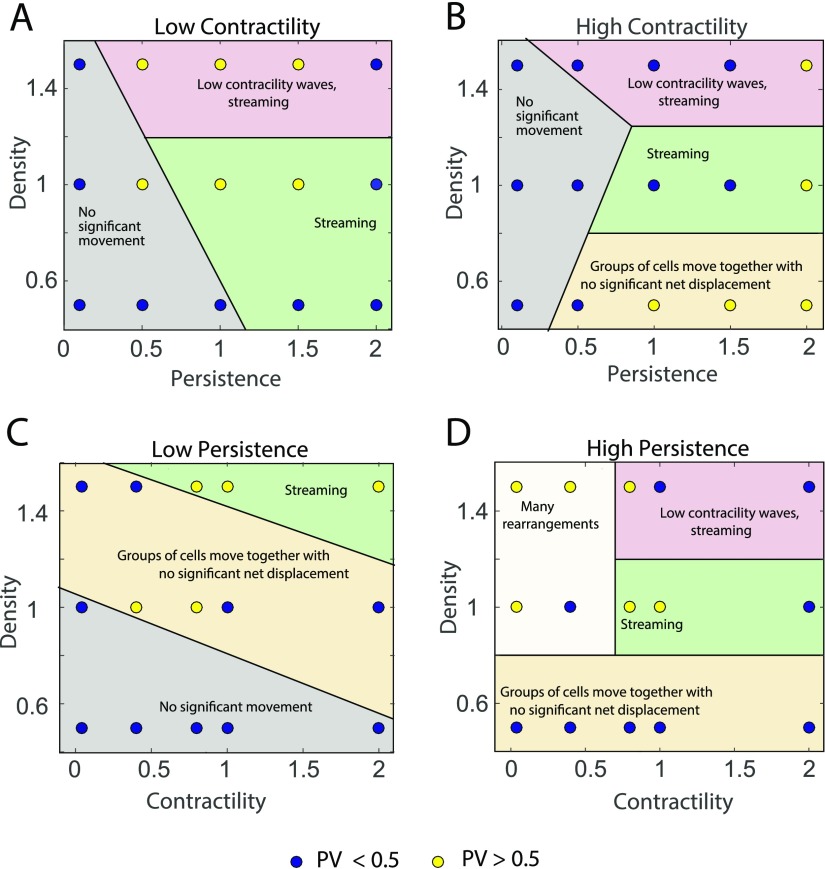
Behavior of the correlation plateau value as a function of cell density, contractility, and cell persistence forces. Correlation plateau value—low (blue), high (yellow)—at low, medium and high densities shown at different persistence and contractility parameter values.

**TABLE I.** Model parameter ranges used for altering persistence, contractility and cell number density in Figs. 3 and 4.

Parameter	Range	Meaning
*k*	0.01-2	Contractility constant
*α*	0.01-2	Persistence force strength
*df*	0.5-1.5	Density factor

The plots in Figs. 3 and 4 show correlation length and plateau values in the modeled epithelial monolayer as a function of persistence and density at low and high contractility and as a function of contractility and density at low and high persistence within the ranges considered.

For the correlation length phase plots (Fig. 3), a value of 7 cell radii is used as a cut-off to define high or low, shown as blue or yellow dots, respectively. Each dot on the plot represents the mean value over three simulations for each condition. For the correlation plateau value phase plots (Fig. 4), a value of 0.5 is used as the cut-off. In both these plots, the mid-high range of values for correlation length or plateau value show a higher order and streaming behavior. The low-mid range values show jammed behavior with a higher probability.

The shaded regions in the plots show different qualitative behavior. We categorize these behaviors into five types.

**FIG. 5.**
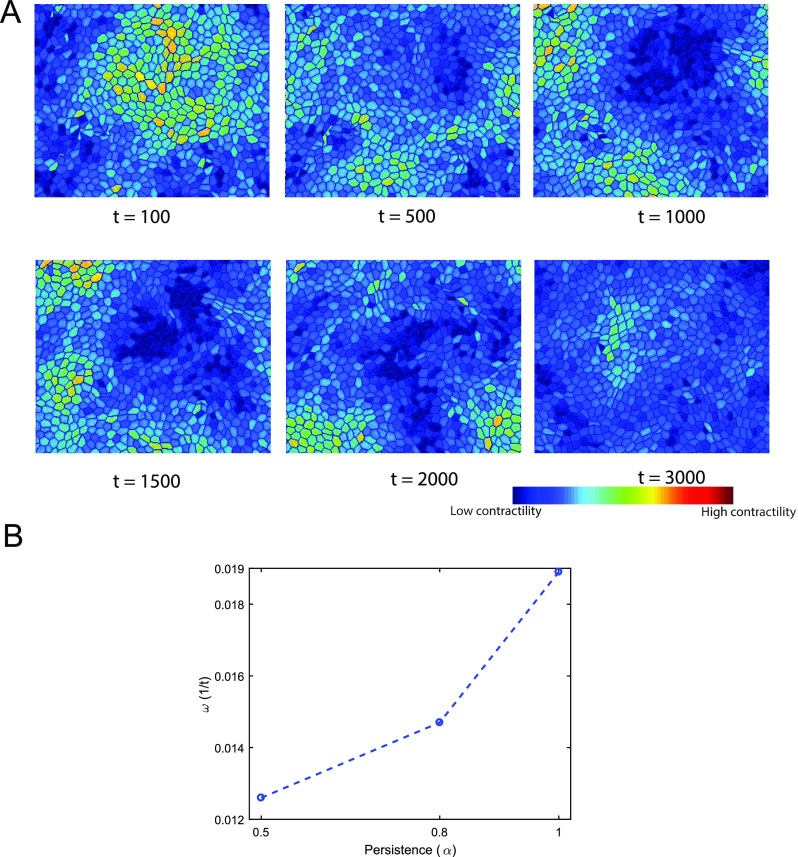
Waves in myosin content. (A) At high persistence forces and medium to high contractility, cells show contractile waves and spatial patterns in myosin content. This is only possible if there is active biochemical signaling regulating myosin contractility. (B) Mean wave frequency (*ω*) increases with the increase in persistence force (*α*). Here, *ω* is obtained by performing a wavelet transform^67^ of the active myosin content *m*(**r**, *t*). The largest wavelet frequency is defined as *ω*.

1. Streaming is defined as the phenomenon where cells move together in the same direction.
2. Streaming with waves in contractility: Myosin content in streaming cells shows wave like patterns over time as shown in Fig. 5 and SM1 (supplementary material movie).
3. No significant movement: Cells hardly move; jammed behavior.
4. Groups of cells moving together with no significant net displacement: Cells move as a group but show random motion with little or no persistent motion.
5. Many rearrangements: Cells undergo many T1 transitions with neighboring cells but no streaming motion.

Continuous cell streaming was observed at medium contractility and high persistence for medium to high cell densities. At high densities, waves in cell myosin content are observed at high persistence forces and medium to high contractile forces. These waves seem to spontaneously arise and disappear. This is not due to box-size effects, since we have seen the same behavior for bigger simulation boxes. The spatial wavelength is independent of the box size. Groups of cells move together without significant net displacement at low densities and medium or high contractile forces. The distance moved by the group increases with the increase in persistence. Jamming or no movement at all is seen at low densities when the persistence is low. As persistence increases, with lower contractile forces cells show lot of rearrangements without any net motion. These results are consistent with model predictions without active cell forces described earlier.^68^

### D. Rotation of cells on a circular ring substrate

Cells migrating in physiological conditions experience various degrees of confinement either because of the extra cellular matrix acting as a boundary or by neighboring cells on multiple sides.^69^ To understand cell motion in the presence of spatial constraints, several studies examined cell behavior by confining single or multiple cells to micropatterned islands of different geometries. Huang *et al.*^70^ showed that when two or three cells are confined to mm scaled fibronectin islands of circular or square geometry, they exhibit spontaneous symmetry breaking and coherent rotation. This phenomenon has also been seen with large numbers of cells (800–10 000 cells per mm^2^).^71^ This type of rotation called as coherent angular motion has shown to be present during the morphogenesis of mammary gland acini and could be important for development.^72^ Albert and Schwarz^73^ show that adhesive micropatterns have a strong influence on collective cell dynamics. Using a two dimensional cellular potts model with cell division, they describe swirl formation on circular and pacman patterns. Simulations show that cells move randomly at first, but their movement becomes swirl like at higher densities. Segerer *et al.*^31^ found that the onset and persistence of coherent angular motion in cells confined in circular micropatterns is a function of number of cells (2–8 cells). They also used a cellular potts model to reproduce the emergence of vortex states.

Recent experiments by Wan *et al.*^74^ have shown that cells on a ring substrate show counter rotation at the inner and outer boundaries. In addition, vortex formation in collective cell migration in narrow channels has also been reported.^75,76^ However, we do not have a good understanding of how cell mechanics and biochemical signaling give rise to such behavior. Previously, a minimal cell centered model incorporating cell geometry and mechanics has been able to show rotation on circular substrates.^24^ In this section, we use the vertex model to look at collective cell behaviors on geometries with two boundaries such as a circular ring.

**FIG. 6.**
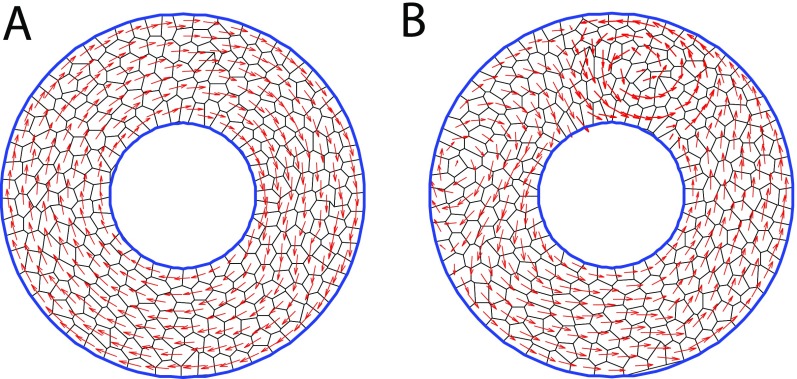
Types of collective motion seen in cells on a ring geometry. 300 cells modeled as polygons on a circular ring substrate. The ratio of inner to outer ring radius is 2:5. Blue circles represent the inner and outer boundaries beyond which the cells are not allowed to move. Cell velocity vectors are shown in red. (A) Coherent rotation and (B) Rotation with vortices observed on ring substrates.

Using our vertex model, we examined model results for cells confined to a ring shaped substrate as shown in Fig. 6. We compare two contractility models discussed earlier—one where the contractility is independent of signaling and the other with a signaling pathway for active contractile force. In the first case, the contractility coefficient is a constant and there is no signaling involved in the model. In the second case, we use the Rho-MLC pathway and make the contractility coefficient proportional to the amount of active myosin. In both these cases, two types of collective cell motions are seen on ring substrates—rotation or rotation with vortex formation (Fig. 6).

#### 1. Vortex formation in the constant contractility coefficient model

**FIG. 7.**
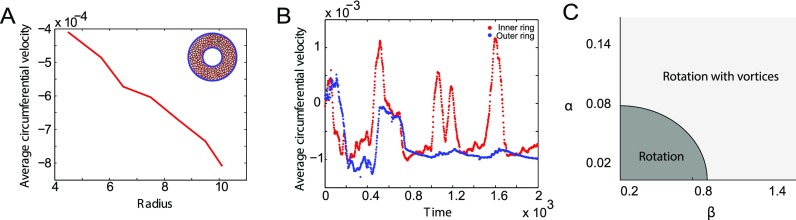
Vortex formation in cells on ring substrates. (A) Plot showing the average circumferential velocity as a function of ring radius. Positive velocity implies clockwise direction and negative velocity implies counter clockwise direction. Mean circumferential velocity is higher at the outer ring when compared to the inner ring. This is expected in coherent rotation when all the cells move together as a solid body with a constant angular velocity. (B) Plot showing the average circumferential velocity as a function of time. Red circles indicate mean velocity at the inner boundary, and blue circles indicate mean velocity at the outer boundary. The mean velocity at the inner boundary shows positive jumps showing clock wise rotation when the velocity at the outer boundary is in the counter clock wise direction. This is indicative of vortex formation as seen in the vortex in (A, inset) where the cells at the inner ring are moving in a direction opposite to the cells at the outer ring. (C) Phase space of persistent force parameters *α* and *β* showing the range of parameters in which vortex formation is seen.

In addition to coherent rotation that is also seen on circular substrates, cells on rings also show the formation of vortices. These vortices span the width of the ring and are dynamic in space and time. Since there is coherent rotation, on an average, all the cells move with the same angular velocity. Hence, the mean circumferential velocity over all cells at long times scales with the radius [Fig. 7(A)]. Plotting mean circumferential velocities at the inner and the outer boundaries as a function of time reflects the formation of vortices [Fig. 7(B)]. Here, the inner ring cells move in the opposite direction as compared to cells in the outer ring intermittently. When there is no vortex formation during rotation, the circumferential velocities at the inner and outer rings move together as shown in Fig. S2.

The ratio of the magnitudes of persistent force to random force determines whether or not cells exhibit coherent angular motion on ring substrates as seen in models of cells on circular substrates.^24^ We also examined the parameter space of the strength of persistent force *α* and the memory decay rate *β* to see what causes the additional complexity of vortex formation during rotation. The range of *α* and *β* values that show rotation with vortices is shown in Fig. 7(C).

#### 2. Model with signaling shows non uniform myosin distribution in cells on a ring

**FIG. 8.**
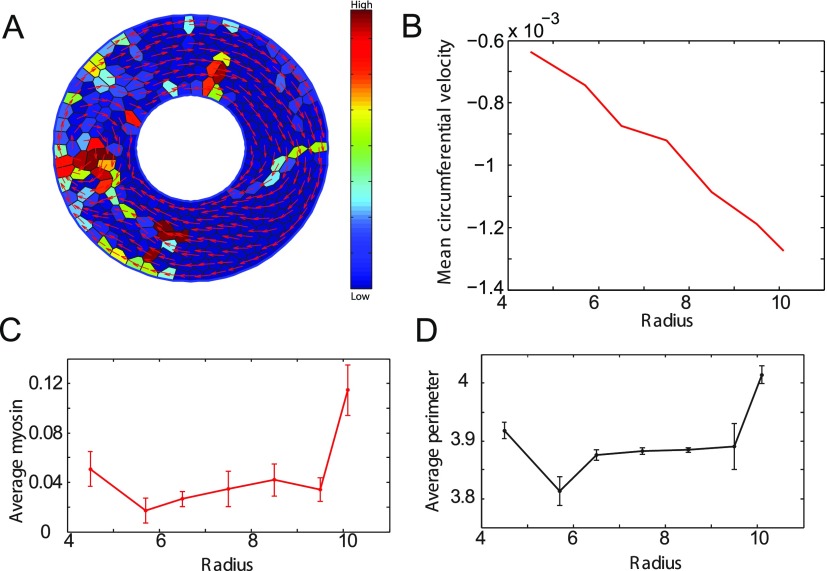
Vortex formation and myosin distribution in cells on ring substrates. (A) Figure showing rotation with vortices on a ring using model with signaling. Velocity vectors are shown in red. The cell colors represent activated myosin content, blue to red showing low to high values. (B) Plot showing the average circumferential velocity as a function of time. Red circles indicate the mean velocity at the inner boundary, and blue circles indicate the mean velocity at the outer boundary. The mean velocity at the inner boundary shows positive jumps showing clock wise rotation when the velocity at the outer boundary is in the counter clockwise direction. This is indicative of vortex formation as seen in the vortex in (A) where the cells at the inner ring are moving in a direction opposite to the cells at the outer ring. (C) Plot showing the average myosin content as a function of ring radius. Higher myosin content in the outer ring cells when compared to the inner ring cells. This trend is also reflected in the plot (D) showing average perimeter as a function of radius.

In the above-mentioned version of the model, the contractile force of a cell was independent of its size or shape. To incorporate the interdependence of cell shape and biochemical signaling, we introduced cell stretch dependent myosin activation into our vertex model to examine motion of cells on ring geometries. This model with signaling also shows coherent angular motion as well as vortex formation as seen in Figs. 8(A) and 8(B). This vortex propagates in space and time around the ring as shown in Fig. S1.

We also compute the distribution of mean myosin content and average cell perimeter as a function of radius [shown in Figs. 8(C) and 8(D)]. The mean myosin content at the edges of ring seems to be about 20% higher than the myosin content in interior. This is consistent with the cell perimeter distribution in the ring.

## III. DISCUSSION

Cell mechanical behavior plays an important role in collective motility of a group of cells. Since cells are active mechanical objects, active changes in the cell mechanical behavior will further modulate the overall behavior of the collective. In this paper, we incorporate known contractile response of cells to external mechanical forces and show that this changes global observable features during collective cell migration. We observe a stronger relationship between the cell velocity and cell density. We also see nonlinear phenomena such as contractile waves and vortex formation when active signaling is incorporated. The active signaling mechanism also explains counter rotations observed for cells migrating on a ring-like confined substrate. These emergent collective phenomena mirror similar types of collective motions in bird flocks,^77^ fish schools,^78^ bacterial waves,^79^ and models of actively propelled particles.^80,81^

In general, all of the mechanical parameters are potentially under biochemical signaling regulation on the time scales of hours. Parameters such as cell area stiffness, *K*, cell-cell adhesion energy Λ, cell preferred volume *A*_0_, and cell persistence force are all regulated by different biochemical pathways. Since most cells migrate appreciably on the orders hours, active cell signaling and changes in protein expression must strongly influence many features of collective cell migration. This paper computationally incorporates one such well known signaling pathway. We also explored how the observed dynamics change when the persistence force is changed. Other pathways such as cell volume and pressure regulation are less clear and should be eventually considered. Cell death and division, although not considered in this modeling effort, are important to understand collective cell motility on a much longer timescale. Cytokinesis, the final step of cell division, also requires crosstalk between several biochemical and mechanical pathways.^82^ Myosin II is recruited to the contractile ring,^83^ and this efficiently drives cell division. Incorporating cytokinesis and the role of myosin in its regulation^84^ could be potentially explored using this model.

This work also highlights the important role of active force generation by cells in organizing collective migration. Passive mechanics of the epithelium is important, but most relevant features in the velocity correlation function are determined by active forces such as persistence and contractility. This is summarized in Figs. 3 and 4, which show rich phase behavior in the velocity correlation function. Within the vertex model formalism, it is straightforward to simulate large number of cells and examine wide parameter ranges. It is also possible to extend the model formalism to 3D and incorporate interaction with external matrix. Different types of cells can be incorporated by selecting individual cell specific parameter sets. Active behavior of the epithelium can be rigorously explored by our model in a variety of settings.

## IV. METHODS

No ethics approval is required since this is a purely modeling paper. Subsections IV A–IV G describe the construction of the vertex model used.

When considering a two dimensional cross section of an epithelial layer, cells can be modeled as polygons, each identified by a set of vertices as shown in Fig. 1. The motion of the vertices determines the motion of the cell. A single vertex is shared by three cells and an edge is shared by two cells. A large system or a periodic system with *N* cells contains 2*N* vertices. A simple argument is as follows: Let us say, each cell has on average ⟨n⟩ vertices. Since each vertex is shared by three cells, the total number of vertices, $N_{\text{vertex}}=N\langle n\rangle /3$. Each edge is shared by two cells, so the total number of edges in the system, $N_{\text{edge}}=N\langle n\rangle /2$. The Euler relation requires that *N* + *N_vertex_* – *N_edge_* = 1, which gives

$$N\left(1+\frac{\left\langle n\right\rangle }{3}-\frac{\left\langle n\right\rangle }{2}\right)=1,$$ (2)

leading to

$$1-\frac{\left\langle n\right\rangle }{6}=\frac{1}{N}.$$ (3)

In the limit of a large system or a periodic system, 1/*N* approaches 0 and hence ⟨n⟩=6. So, we have $N_{\text{vertex}}=N\langle n\rangle /3=2N$. This tells us that the number of total vertices is known if we know how many cells the system contains. This property can be used to generate the initial distribution of vertices.

### A. Equation of motion of a cell vertex

Forces acting on a cell vertex can be classified into passive, active, or frictional. At cellular length scales and time scales, inertia is negligible. Hence, mechanical forces are balanced by overdamped frictional forces, and an equation of motion can be written for the *i*-th cell vertex

$$\underset{cell-substrate\;friction}{\underset{︸}{\eta_{s}\frac{dr_{i}}{dt}}}+\underset{cell-cell\;friction}{\underset{︸}{F_{fc,i}}}=\underset{passive\;force}{\underset{︸}{F_{p,i}}}+\underset{active\;force}{\underset{︸}{F_{a,i}}},$$ (4)

where *η_s_***v**_*i*_ is the friction between the cell and substrate (viscous drag), *η_s_* is the frictional coefficient, $v_{i}=\frac{dr_{i}}{dt}$ is the velocity of vertex *i*, and **r**_*i*_ is its position. **F**_*fc*__,__*i*_ is the friction between cells, and **F**_*p*__,__*i*_ is the passive force arising from cell deformation and cell-cell adhesion. **F**_*a*__,__*i*_ denotes the active force arising from cell contractility and persistent protrusive forces.

### B. Passive force

Eukaryotic cells resist mechanical deformation primarily because cells actively maintain their volume on short time scales (∼seconds) using ion channels and pumps. This mechanism controls cells' internal osmotic and hydrostatic pressures and their water content.^44,56^ In addition, adhesion between cells in a sheet, e.g., due to cadherin bonds, results in contact surface energy with neighboring cells. Such passive forces can be calculated from an energy (*U_p_*) of the form

$$U_{p}=\sum_{J=1}^{N}\frac{K}{2}{\left(A_{J}-A_{0}\right)}^{2}+\sum_{i,j}\Lambda l_{ij},$$ (5)

$$F_{p,i}=-\frac{\partial U_{p}}{\partial r_{i}}.$$ (6)

Here, *K* is an effective modulus of the cell that describes resistance of cells to volume changes, *A_J_* is area of cell *J*, *A*_0_ is the preferred cell area which could depend on the cell type, Λ is the adhesion energy per unit length, and *l_ij_* is the edge length between vertex *i* and *j*.

### C. Active force

In addition to passive mechanics, cells also generate active forces. These could be from the intrinsic contractility of a cell due to molecular motors or cell's protrusions in the polarization direction. In this model, we consider three kinds of active forces—contractile force, persistent force, and a random force due to polarization diffusion

$$F_{a,i}=\underset{Contractile\;force}{\underset{︸}{F_{c,i}}}+\underset{Persistent\;force}{\underset{︸}{F_{p,i}}}+\underset{Random\;force}{\underset{︸}{F_{R,i}}}.$$ (7)

#### 1. Contractile force

Phosphorylated myosin leads to contractile forces in the cell cortex. Taking this into account, the contractile energy function (*U_c_*) is assumed to be

$$U_{c}=\sum_{J=1}^{N}\frac{T_{J}}{2}L_{J}^{2},$$ (8)

$$T_{J}=kM_{J},$$ (9)

where *L_J_* is the perimeter of cell *J* and *T_J_* is the contractility coefficient that is proportional to the amount of phosphorylated myosin, and *M_J_* in a cell which in turn is determined by the Rho-ROCK-myosin signaling pathway described below. The contractile force is then $F_{c,i}=-\frac{\partial U_{c}}{\partial r_{i}}$.

#### 2. Rho-ROCK-myosin signaling pathway

Studies suggest that when a cell is subjected to an external stretch, the activation of RhoGTPase increases.^46^ This ultimately leads to phosphorylation of myosin which then tries to contract the cell,^52^ resulting in a negative feedback loop. Although this pathway involves several other signaling molecules, we use a simplified version of the pathway in this model. The simplified pathway involves two components—Rho and Myosin but captures the underlying phenomenon. It is built into the model as a system of ordinary differential equations (ODEs) for fractions of activated Rho and Myosin. The fraction of activated RhoGTPase obeys the following ODE:

$$\frac{d\rho_{J}}{dt}=G_{\rho }H(s_{J})\frac{s_{J}^{n}}{K_{s}+s_{J}^{n}}(1-\rho_{J})-D_{\rho }\rho_{J},$$ (10)

where *H* is the Heaviside step function making sure *ρ* gets activated only in response to cell stretch, *G_ρ_* is the maximum activation rate of *ρ_J_*, *s_J_* = *L_J_* – *L*_0_ is the stretch of the cell, *L*_0_ being the preferred perimeter corresponding to the preferred area *A*_0_, *K_s_* is the half maximal response constant, *n* is the hill coefficient, and *D_ρ_* is the deactivation rate of *ρ*. The fraction of phosphorylated myosin follows a similar kinetic equation:

$$\frac{dM_{J}}{dt}=G_{M}\rho_{J}(1-M_{J})-D_{M}M_{J},$$ (11)

where *G_M_*, *D_M_* are activation and deactivation rates of myosin. Here, we assume uniform spatial distribution of Rho or myosin within each cell. In reality, this is not true and there could be nonuniform distribution of these molecules within the cell leading to polarization. It is also possible to implement models of cell polarization in this vertex model.^57^ In addition, the contractile force is assumed to originate from the junctional regions of the cell and hence the contractile energy is a function of the cell perimeter. In reality, medial myosin accumulation can also lead to contraction,^58^ which is not considered in this model.

#### 3. Persistent force

Cells migrate in a directed fashion over a characteristic time required to disassemble and reassemble cytoskeletal networks necessary for motility.^59,60^ Persistent force in our model comes from the ability to move in a certain direction persistently before making a turn. It is described phenomenologically as a term that depends on cells' past velocities

$$F_{p,i}=\alpha \frac{\int_{-\infty }^{t}\;\text{exp}\;(-\beta (t-t\prime ))v_{i}(t\prime )dt\prime }{\left|\int_{-\infty }^{t}\;\text{exp}\;(-\beta (t-t\prime ))v_{i}(t\prime )dt\prime \right|},$$ (12)

where *α* is the strength of the persistent force, *β* is a constant that determines the decay rate of persistence, and $v_{i}=\frac{dr_{i}}{dt}$ is the velocity of the vertex *i*. The denominator is a normalization factor. For large *β*, this term is also equivalent to the persistent part in the persistent random walk model,^24,59^ which has been used to describe single cell motility.

#### 4. Random force

Random force due to polarization fluctuation is modeled as Gaussian white noise with zero mean and finite variance satisfying the following relations:

$$\langle F_{R,i}\rangle =0,$$ (13)

$$\langle F_{R,q}(t)\cdot F_{R,s}(t\prime )\rangle =\sigma^{2}\delta (t-t\prime )\delta_{qs},$$ (14)

where *σ* is the magnitude of variance characterizing magnitude of polarization fluctuation, and *δ*(*t*), *δ_qs_* are Dirac's and Kronecker's *δ*-functions, respectively. In reality, there is very little information regarding polarization fluctuation statistics in migrating cells, and further information would help in developing a better quantitative description.

### D. Friction

#### 1. Cell-cell friction

**FIG. 9.**
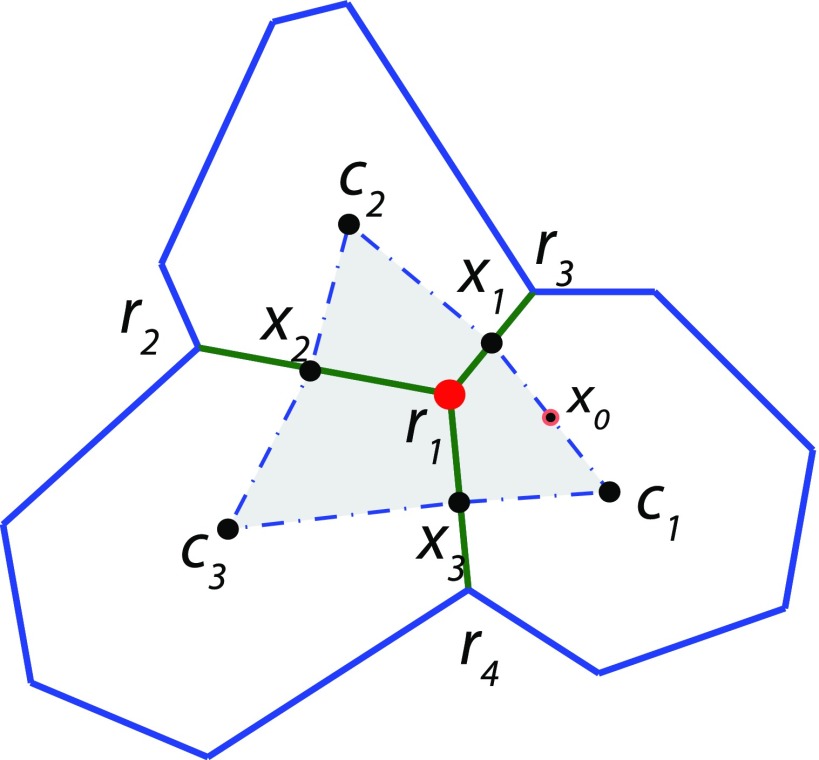
Finite volume method for computing cell-cell friction. Frictional forces between cells in the sheet can be calculated from shear stress within the volume element surrounding the vertex. The procedure to compute the friction force acting on vertex **r**_1_ is described in the text [Eqs. (15)–(28)].

Considering the part fluid like behavior of the epithelial sheet, friction between cells can be calculated from the in plane shear stress. This is computed using a finite volume approach. The cell-cell friction force experienced by a vertex is defined as the total shear force on the volume element^61^ defined by the cell centers neighboring the vertex and the midpoints of neighboring edges as shown in Fig. 9. Deviatoric stress, which is the total stress acting on a volume element minus the hydrostatic stress, is given by Eq. (15). Frictional force is obtained by integrating this stress over the volume element

$$\sigma_{s}=\eta_{c}\left(\nabla v+{\left(\nabla v\right)}^{T}-\frac{2}{3}\nabla \cdot vI\right),$$ (15)

$$F_{fc,i}=\oint \sigma_{s}\cdot ndS,$$ (16)

where *η_c_* is the cell-cell friction coefficient, ∇**v** is the velocity gradient within the volume element, *S* is the surface enclosing the volume element, and **n** is the outward directed normal to the volume. A simplification is to assume the epithelium is incompressible, so that ∇⋅ *v *=* *0, giving

$$\sigma_{s}=\eta_{c}(\nabla v+{\left(\nabla v\right)}^{T}).$$ (17)

Substituting this simplified form into the friction force equation, Eq. (16) gives

$$F_{fc,i}=\eta_{c}\oint \nabla v\cdot ndS.$$ (18)

Decomposing **v** and **F**_*fc*_ into *x* and *y* components, friction force can be written as

$$F_{fc,x}=\eta_{c}\oint \left(\frac{\partial v_{x}}{\partial x}n_{x}+\frac{\partial v_{x}}{\partial y}n_{y}\right)dS,$$ (19)

$$F_{fc,y}=\eta_{c}\oint \left(\frac{\partial v_{y}}{\partial x}n_{x}+\frac{\partial v_{y}}{\partial y}n_{y}\right)dS.$$ (20)

The velocity field can be computationally determined by a Taylor series expansion of the functions *v_x_* and *v_y_* about the midpoint of each edge of the volume element. Using the edge *c*_1_*x*_1_ with midpoint *x*_0_ as an example, the expansion is

$$v_{x}(x)\approx v_{x_{0}}+(x-x_{0}){\frac{\partial v_{x}}{\partial x}|}_{x_{0}}+(y-y_{0}){\frac{\partial v_{x}}{\partial y}|}_{x_{0}},$$ (21)

$$v_{y}(x)\approx v_{y_{0}}+(x-x_{0}){\frac{\partial v_{y}}{\partial x}|}_{x_{0}}+(y-y_{0}){\frac{\partial v_{y}}{\partial y}|}_{x_{0}},$$ (22)

where the nodes *c*_1_, *r*_1_, and *r*_3_ are used to obtain

$$v_{x_{0}}=\frac{2v_{x}(c_{1})+v_{x}(r_{1})+v_{x}(r_{3})}{4},$$ (23)

$${\frac{\partial v_{x}}{\partial x}|}_{x_{0}}=\frac{r_{1y}[v_{x}(r_{3})-v_{x}(c_{1})]+r_{3y}[v_{x}(c_{1})-v_{x}(r_{1})]+c_{1y}[v_{x}(r_{1})-v_{x}(r_{3})]}{r_{1y}(r_{3x}-c_{1x})+r_{3y}(c_{1x}-r_{1x})+c_{1y}(r_{1x}-r_{3x})},$$ (24)

$${\frac{\partial v_{x}}{\partial y}|}_{x_{0}}=\frac{r_{1x}[v_{x}(r_{3})-v_{x}(c_{1})]+r_{3x}[v_{x}(c_{1})-v_{x}(r_{1})]+c_{1x}[v_{x}(r_{1})-v_{x}(r_{3})]}{r_{1y}(r_{3x}-c_{1x})+r_{3y}(c_{1x}-r_{1x})+c_{1y}(r_{1x}-r_{3x})},$$ (25)

and

$$v_{y_{0}}=\frac{2v_{y}(c_{1})+v_{y}(r_{1})+v_{y}(r_{3})}{4},$$ (26)

$${\frac{\partial v_{y}}{\partial x}|}_{x_{0}}=\frac{r_{1y}[v_{y}(r_{3})-v_{y}(c_{1})]+r_{3y}[v_{y}(c_{1})-v_{y}(r_{1})]+c_{1y}[v_{y}(r_{1})-v_{y}(r_{3})]}{r_{1y}(r_{3x}-c_{1x})+r_{3y}(c_{1x}-r_{1x})+c_{1y}(r_{1x}-r_{3x})},$$ (27)

$${\frac{\partial v_{y}}{\partial y}|}_{x_{0}}=\frac{r_{1x}[v_{y}(r_{3})-v_{y}(c_{1})]+r_{3x}[v_{y}(c_{1})-v_{y}(r_{1})]+c_{1x}[v_{y}(r_{1})-v_{y}(r_{3})]}{r_{1y}(r_{3x}-c_{1x})+r_{3y}(c_{1x}-r_{1x})+c_{1y}(r_{1x}-r_{3x})}.$$ (28)

Analogous equations for the edges *x*_1_*c*_2_, *c*_2_*x*_2_, *x*_2_*c*_3_, *c*_3_*x*_3_, and *x*_3_*c*_1_ can be derived to compute the entire path integral and hence the friction force between cells.

#### 2. Substrate drag force

The substrate drag force **F**_*cs*__,__*i*_ is defined as the friction between the cell *i* and the substrate (including the media around it) and is assumed to be proportional to the cell velocity **v**_*i*_. The coefficient of friction *η_s_* is assumed to be a constant

$$F_{cs,i}=\eta_{s}v_{i}.$$ (29)

### E. Topology changes

**FIG. 10.**
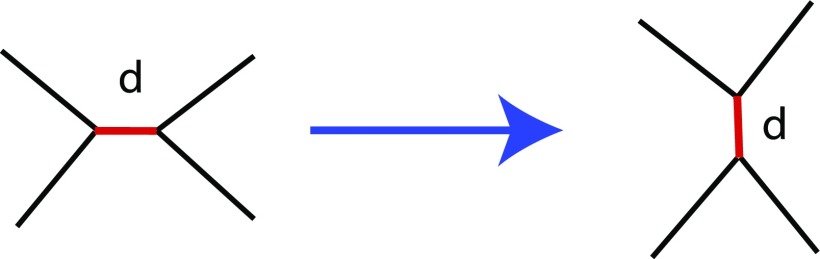
Topology changes. T1 transition, a neighbor exchange method to which allows changes in connectivity of vertices and allows cells to make and break bonds with neighbors.

To faithfully represent collective cell behavior, we need to allow cells to change their neighbors. Topological changes, i.e., changes in connectivity of vertices, occur through T1 transitions. T1 transition is a neighbor exchange method which allows cells to make and break edges with its neighbors. If the cell edge length goes below a certain threshold, T1 transition is allowed only if after the transition the two vertices of the edge move away from each other. The edge undergoing transition is rotated by 90° around its midpoint as shown in Fig. 10. Additionally to maintain the integrity of the two dimensional system, restrictions are placed on the movements of vertices to ensure that edges do not cross or cells do not fold onto themselves.

### F. Implementation

This model is implemented in both MATLAB and Fortran. All the parameters used in the model are listed in Table II. Parameters are rendered dimensionless using a length scale of $\sqrt{A_{0}}$, time scale of $\frac{\eta_{s}}{KA_{0}}$, and an energy scale of $KA_{0}^{3/2}$.

**TABLE II.** Model parameters used in the simulations.

Parameter	Value	Meaning
*N*	1000	Number of cells in simulation
*η_s_*	1	Cell-substrate friction coefficient
*η_c_*	0.1	Cell-cell friction coefficient
*K*	5	Area elastic modulus
*A*_0_	1	Preferred area
Λ	0.5	Cell-cell adhesion strength
*k*	2	Contractility constant
*α*	0.1	Persistence force strength
*β*	0.1	Persistence force decay
*G_ρ_*	0.5	Maximal activation rate for *ρ*
*D_ρ_*	0.1	Inactivation rate for *ρ*
*G_M_*	0.5	Maximal activation rate for M
*D_M_*	0.05	Inactivation rate for M
*K_s_*	120	Half maximum response constant
*n*	2	Hill coefficient
*t*1	0.07	T1 transformation threshold

### G. Radial correlation function

To quantitatively measure organizational features of the migrating collective, we examine a radial spatial correlation function for the simulated cell velocity fields. Cell motion is aligned if the value of this function is one and it is random if the value is zero. Defining this function at various radial distances gives us an idea of local and broader regional organization. The radial correlation function for cell velocities at a distance *ρ* is defined as

$$C(\rho )=\frac{\sum_{ij}C_{ij}I_{ij}}{\sum_{ij}I_{ij}},$$ (30)

where the sum is over all cell vertices. Here, *C_ij_* is a correlation matrix between vertex velocities **v**_*i*_ and **v**_*j*_ and is defined as

$$C_{ij}=\frac{v_{i}\cdot v_{j}}{\left\|v_{i}\right\|\left\|v_{j}\right\|},$$ (31)

and *I_ij_* is an indicator matrix that takes on a value of 1 when the vertex pair belongs to a radial annulus of diameter *ρ* and 0 otherwise

$$I_{ij}=\{\begin{array}{c} 1,\;\text{if}|r_{i}-r_{j}|\;\in \;\rho \\ 0,\;\text{otherwise}. \end{array}$$ (32)

## SUPPLEMENTARY MATERIAL

See supplementary material for additional figures on vortex propagation in cell velocities when cells are confined to a ring structure and for a movie on collective cell streaming with wave like patterns in myosin content.
